# Dependency parsing of biomedical text with BERT

**DOI:** 10.1186/s12859-020-03905-8

**Published:** 2020-12-29

**Authors:** Jenna Kanerva, Filip Ginter, Sampo Pyysalo

**Affiliations:** TurkuNLP Group, University of Turku, Turku, Finland

**Keywords:** Parsing, Deep learning, CRAFT

## Abstract

**Background:**

Syntactic analysis, or parsing, is a key task in natural language processing and a required component for many text mining approaches. In recent years, Universal Dependencies (UD) has emerged as the leading formalism for dependency parsing. While a number of recent tasks centering on UD have substantially advanced the state of the art in multilingual parsing, there has been only little study of parsing texts from specialized domains such as biomedicine.

**Methods:**

We explore the application of state-of-the-art neural dependency parsing methods to biomedical text using the recently introduced CRAFT-SA shared task dataset. The CRAFT-SA task broadly follows the UD representation and recent UD task conventions, allowing us to fine-tune the UD-compatible Turku Neural Parser and UDify neural parsers to the task. We further evaluate the effect of transfer learning using a broad selection of BERT models, including several models pre-trained specifically for biomedical text processing.

**Results:**

We find that recently introduced neural parsing technology is capable of generating highly accurate analyses of biomedical text, substantially improving on the best performance reported in the original CRAFT-SA shared task. We also find that initialization using a deep transfer learning model pre-trained on in-domain texts is key to maximizing the performance of the parsing methods.

## Background

The task of automatically analyzing raw text to determine the syntactic structure of input sentences and generating representations of those structures in some established formalism is known as *syntactic analysis* or *parsing*. Parsing is a core task in natural language processing (NLP) and a required component of many information extraction and text mining systems, which make use of syntactic structures to determine e.g. which relations involving specific named entities, such as protein-protein interactions, are stated in text. Parsing research was for long dominated by *constituency* (or *phrase structure*) formalisms due in part to the influence of resources such as the Penn Treebank [1] and tools such as the Stanford [2] and BLLIP [3] parsers. However, many systems making use of syntactic analyses for information extraction tasks in biomedicine [4–6] as well as in other domains [7, 8] have preferred *dependency* representations of syntax, which capture relations between words more explicitly [9, 10]. In recent years, there has been a considerable shift toward dependency representations also within parsing research, driven in part by the success of Universal Dependencies (UD), a broad collaborative project to introduce cross-linguistically consistent dependency annotation for many languages [11, 12]. The UD effort has to date led to the introduction of more than 150 treebanks in 90 languages (https://universaldependencies.org/) and its resources served as the basis of the popular Conference on Computational Natural Language Learning (CoNLL) shared tasks on multilingual dependency parsing in 2017 and 2018 [13, 14]. While the UD effort and these tasks have served to substantially advance the available resources and the state of the art in highly multilingual dependency parsing, there has been comparatively little effort focusing on dependency parsing for specialized domains such as biomedicine. In 2019, a shared task on biomedical dependency parsing was organized as the CRAFT-SA (Structural Annotation) subtask in the CRAFT shared tasks [15], a set of community challenges building on the data of the Colorado Richly Annotated Full Text (CRAFT) corpus [16, 17]. Our group (TurkuNLP) participated in this task, achieving the highest performance in the task [18]. We build further on the data and other resources of the shared task in this paper, applying models and methods reflecting the latest developments in neural dependency parsing.

Along with an increased focus on dependency representations of syntax, there have recently been notable methodological shifts in parsing, mirroring general trends in machine learning. First, methods have moved from statistical approaches [2, 19, 20] and machine-learning approaches building on explicitly defined features [21–24] toward deep neural methods employing dense features learned from data [25–27]. In an associated trend, there has been substantial recent interest on transfer learning, which in the context of this paper refers to using models pre-trained on large unannotated text corpora, and subsequently fine-tuned for the specific task at hand. Initially, the focus was on shallow approaches generating context-free representations of word meaning, such as word2vec [28] and GloVe [29], and in the last few years increasingly on deep contextualized models of meaning such as ULMFiT [30], ELMo [31], and BERT [32]. Of these, the BERT model has been particularly influential, notably advancing on the state of the art in several NLP tasks [33] and serving as the basis for many recent studies in deep transfer learning [34, 35]. The best-performing system in the CoNLL 2017 shared task was a deep learning model using context-free word representations induced from billions of words of raw text [36]; in 2018, many CoNLL participants built on this approach, including in a top-performing system for many metrics specifically through integrating information from deep contextualized word vectors [37]. In the original CRAFT-SA shared task, we participated with the Turku Neural Parser Pipeline [38], a retrainable full parser pipeline based on the winning CoNLL’17 parser [36] and a top ranking system in CoNLL’18. In this paper, we extend on our previous work in two primary ways: (1) we replace a substantial part of the parser pipeline with the recent deep neural parser UDify [39], which is based on the BERT model and, (2) we explore a broad range of alternative BERT models to use for initializing UDify, replacing the multilingual model that the parser uses by default. We demonstrate that both of these modifications substantially improve on the best performance achieved at the original shared task, together achieving a 15% reduction in the error rate of the previous state of the art for the standard labeled attachment score (LAS) metric.

In the following, we first introduce the CRAFT-SA task data and the BERT models considered in this study. We then present the baseline approaches and the previous state of the art model from the original shared task, and introduce the updated version of our parsing pipeline proposed for biomedical dependency parsing in this paper. We then present and discuss the results and conclude with a discussion of future work.

## Data

We make use of a single manually annotated resource in this work: the syntactic annotations of the CRAFT corpus. We additionally use a selection of deep language models pre-trained on unannotated texts. We introduce these resources in this section.

**Table 1:** CRAFT corpus structural annotation statistics

	Train	Devel	Test
Documents	57	10	30
Sentences	18,563	3168	9099
Tokens	477,825	83,207	232,619

**Table 2:** BERT model statistics: model parameters, vocabulary size in wordpieces, and number of English language words in the pretraining data

Model	Params (M)	Vocab (K)	Words (Eng.) (B)
Google BERT large	340	29	3.3
Google mBERT	180	120	2.5
SciBERT base scivocab uncased	110	31	3.2
BioBERT large v1.1. custom vocab	360	59	21.3
BlueBERT base P+M	110	31	4.5

**Table 3:** Comparison of $F_1$ results for previously proposed parser variant (TurkuNLP-ST) and newly proposed approach initialized with various BERT models

Metric	Method					
	TurkuNLP-ST	mBERT	BERT-large	BioBERT	BlueBERT	SciBERT
UPOS	98.54	98.75	98.76	**98.79**	97.70	98.72
UFeats	98.63	98.76	98.77	**98.79**	97.54	98.75
Lemmas	99.44	99.44	99.45	**99.46**	99.21	**99.47**
UAS	91.54	92.66	93.16	**93.45**	91.34	92.93
LAS	90.28	91.38	91.97	**92.31**	89.42	91.67
CLAS	87.96	88.80	89.69	**90.04**	86.10	89.20
MLAS	85.93	86.88	87.77	**88.22**	82.76	87.22
BLEX	87.31	88.08	88.98	**89.36**	85.17	88.51

All experiments performed using gold segmentation

Maximum score in bold

**Table 4:** Comparison to previously published results using CRAFT-SA test data with predicted segmentation

Method	Metric		
	LAS	MLAS	BLEX
Baseline	56.68	44.22	0.0
SpaCy	69.32	0.0	54.80
TurkuNLP-ST	89.70	85.55	86.63
Ours	**91**.**21**	**86**.**63**	**87**.**68**

Maximum score in bold

### CRAFT corpus

The CRAFT corpus consists of 97 full-text articles that have been manually annotated for multiple layers of information, including normalized mentions of concepts such as entity names, coreference, and sentence syntax [16, 17]. In this work, we only consider the syntactic annotation of the corpus.

For the purposes of the shared task, the 97 documents of the CRAFT corpus were divided into a visible subset of 67 articles that were made available to participants with full annotation and a blind subset of 30 articles for which annotations were held out and participants were only provided with the raw, unannotated texts of the articles. As there is no pre-defined division of the data into training and development sets, we split the provided visible dataset randomly in terms of documents into 57 training documents and 10 development documents that were used only for early stopping during training. The statistics of this split are shown in Table 1.

**Fig. 1:**
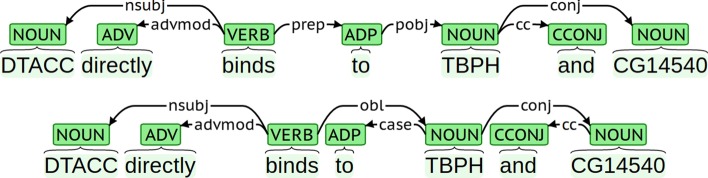
Illustration of Stanford Dependencies (top) and Universal Dependencies (bottom) analyses for an example sentence. The CRAFT dependency annotation follows the former representation. (Example from PMCID:15207008, figure adapted from [18])

The dependency annotation of the data is automatically created by conversion from the Penn Treebank constituency representation [1] used in the CRAFT corpus. This conversion is based on the implementation by Choi and Palmer [40], followed by further custom post-processing by the shared task organizers. The resulting dataset conforms to the CoNLL-U data format, but the syntactic annotation is not fully in line with the Universal Dependencies guidelines [18]. Rather, it more closely resembles the Stanford Dependencies (SD) representation, a predecessor of the Universal Dependencies scheme [41, 42]. Most importantly, while the UD scheme consistently assigns relations between content words, with function words being dependents, this principle is enforced to a lesser degree in SD. A typical difference in the analysis of prepositional phrases is illustrated in Fig. 1. There are also a number of other consistent differences between SD and UD, such as the attachment of coordinating conjunctions to the first conjunct in SD and to the nearest right-hand conjunct in UD.

These differences do not represent complications from the point of view of the parser pipelines considered in this work, which are fully based on machine learning and agnostic to the details of the representation. However, they prevent, or at least make considerably harder, treebank pooling and other techniques that combine multiple resources to improve parsing performance, a limitation we have previously discussed in further detail in our original shared task study [18].

### BERT models

Deep language models, especially recent models based on the Transformer neural network architecture [43] have had a major impact in natural language processing, leading to a new state of the art performance on a large number of established reference tasks. Arguably the model with the broadest impact to date is the BERT model of Devlin et al. [32]. These language models are pre-trained on a large amounts of raw, unannotated text, and subsequently fine-tuned with annotated task-specific data to create models for specific downstream tasks such as parsing. Since pre-training such models frequently involves fitting hundreds of millions of parameters to examples derived from billions of words of text through millions of minibatch training steps at a non-trivial computational cost, pre-trained models are typically distributed publicly, and the ability to choose the correct pre-trained model for the task at hand from among the large and fast-growing set of published models is an important factor for success.

One major difference between pre-trained language models is the text domain from which the pre-training data is drawn, which affects e.g. the vocabulary known to the model. Similarly to how the previous generation of context-free word representations benefit from initialization on in-domain data [44, 45], deep contextual models such as BERT should generally be pre-trained using data that reflects the domains that the models will be fine-tuned for to maximize performance [46–48]. In addition to the pre-training data, the models can also differ in the various training and model size parameters. Two common sizes for BERT models are *Base*, with 12 Transformer layers and approx. 110 million parameters, and *Large*, with 24 layers and approx. 340 million parameters, where the exact parameter count varies based on the vocabulary size. While more demanding of computational resources in pre-training, fine-tuning, and prediction, Large models generally provide for better performance, and we here focus on Large variants of BERT models whenever available.

In order to assess the impact of the choice of the pre-trained model on parsing performance, we here evaluate performance initializing the parser with each of the following BERT models:

*Google BERT Large* a BERT Large model introduced by Devlin et al. [32] trained on 2.5B words of the English Wikipedia and 0.8B words of BooksCorpus [49] texts, this model represented the state of the art in many general English NLP tasks when published.

*Google mBERT* a BERT Base model trained on the Wikipedias of over 100 different languages. This model was used as the basis for fine-tuning in the study introducing the UDify parser that substantially advanced the state of the art in multilingual UD parsing [39].

*SciBERT Base scivocab uncased* a BERT Base model pre-trained by Beltagy et al. [46] on scientific text from the *Semantic Scholar* resource, and one of the first BERT models specifically including biomedical domain scientific publications in its pre-training data.

*BioBERT Large v1.1. custom vocab* a BERT Large model pre-trained by Lee et al. [48] on the combination of English Wikipedia, BooksCorpus, PubMed, and PubMed central texts. Fine-tuning the model was shown to improve on previously published results on several biomedical NLP tasks.

*BlueBERT Base P+M* (previously named NBCI-BERT) a BERT Base model trained on the combination of PubMed abstracts (90% of the pre-training data) and MIMIC-III clinical notes (10% of pre-training data) by Peng et al. [47] and shown to advance the state of the art across a range of NLP tasks in related domains.

The evaluated models thus include two that are pre-trained on “general” language (primarily Wikipedia) and three including scientific domain texts, with BioBERT and BlueBERT specifically targeting the biomedical domain. The models also represent both Base and Large BERT variants. Table 2 summarizes the key statistics of these models. We note that in addition to being pre-trained on the largest corpus among these models, BioBERT has the largest vocabulary size and, hence, as a BERT Large model also the largest number of parameters.

## Methods

We next introduce our parser pipeline, the reference methods, and the evaluation criteria applied in the original CRAFT-SA task as well as in this study.

### Turku Parser

The primary parser used in all experiments as well as in our original Shared Task submission is the Turku Neural Parser Pipeline [38], a full parser pipeline capable of sentence and word segmentation, part-of-speech and morphological tagging, syntactic parsing, and lemmatization. The pipeline thus produces fully parsed, tagged and lemmatized output from a raw, plain text input. The Turku Parser was ranked second on labeled attachment score (LAS) and morphology-aware labeled attachment score (MLAS), and first on the bilexical dependency score (BLEX) metric in the CoNLL-2018 Shared Task [14], first by all primary metrics in the original CRAFT-SA task [15, 18], and first by all primary metrics in the recent IWPT 2020 shared task [50, 51], demonstrating its highly competitive performance. In this study, we integrated a new parsing component into the parser pipeline, replacing the parser of Dozat et al. [36] with the more recent UDify parser [39], enabling us to fine-tune this component on BERT models.

In our revised version of the pipeline, text segmentation is realized using UDPipe, which predicts token and sentence boundaries jointly, using a single-layer bidirectional GRU neural network [52]. Part-of-speech tagging, morphological feature assignment, and dependency parsing are performed jointly using the UDify parser [39]. This parser is primarily based on encoding the input text with the BERT language model encoder, followed by several task-specific prediction layers that carry out tagging and dependency parsing based on the BERT representation. The main strength of the model is in the BERT encoder, as the task-specific layers are comparatively simple. Finally, we use the universal lemmatizer of Kanerva et al. [53], a sequence-to-sequence model where the lemma is generated one character at a time from the given input word form and its morphological features.

The Turku parser pipeline integrates all these individual components into a single system, where each of its components is individually retrainable and in no way restricted to the UD scheme, allowing the pipeline to be easily trained on the CRAFT corpus, even though it departs from the UD representation in various details.

### Reference systems

We compare the performance of our proposed approach to all systems for which performance on the CRAFT-SA task data was reported in the original shared task [15], namely the following:

*Baseline* is a baseline system constructed by the shared task organizers. The system applies the *punct* segmentation method implemented in the Python Natural Language Toolkit (NLTK) library [54] for sentence segmentation and tokenization, and the neural SyntaxNet model [55] for POS tagging and dependency parsing. The baseline does not implement lemmatization.

*SpaCy* a system based on the SpaCy dependency parser [56] was applied by the group identified as T013 in the original CRAFT-SA shared task [15]. While we are not aware of a detailed published description of this system, we provide for reference results for the better of the two runs submitted for this system to the task.

*TurkuNLP-ST* the version of the Turku Neural parser pipeline applied by our group in the original shared task, in which we were identified as T014 [15, 18]. As for the SpaCy system, we repeat here for reference results for our best-performing submission to the original shared task. We also include new results for the system using gold segmentation as a point of comparison for our newly proposed approach.

### Evaluation criteria

To maintain direct comparability with the results of the original CRAFT-SA task, we apply identical criteria for evaluating the performance of the various methods. Performance in the CRAFT-SA task was evaluated using the 2018 version of the CoNLL shared task evaluation script (conll18_ud_eval.py), and performance was compared primarily in terms of the same three metrics as in the CoNLL’18 shared task, namely the labeled attachment score (LAS), the morphology-aware labeled attachment score (MLAS), and the bi-lexical dependency score (BLEX). In brief, these metrics are defined as follows:

*Labeled attachment score (LAS)* is the ratio of tokens for which the syntactic head and the dependency relation are predicted correctly. LAS is a widely-applied standard metric for evaluating the performance of dependency parsers, and we used it as the primary metric for assessing our methods during development.

*Morphology-aware labeled attachment score (MLAS)* is a variation of LAS for content words where in addition to the head and dependency relation also the universal POS tag, selected morphological features, and particular functional dependents must be correctly predicted.

*Bilexical dependency score (BLEX)* is likewise a variation of LAS focused on content words, requiring the lemmas of related words to be correctly predicted in addition to the head and dependency relation.

In addition to these primary metrics, we report performance for metrics assessing the correct prediction of universal part-of-speech tags (UPOS), universal word features (UFeats), the base forms of words (Lemmas), as well as the unlabeled attachment score (UAS), which only evaluates the dependency structure without labels, and the content-word labeled attachment score (CLAS), which disregards functional words whose attachment is comparatively easy to resolve. We refer to the studies introducing these metrics and their use in the shared tasks for full details on the definitions of these established metrics [13–15].

Both the CoNLL and CRAFT shared tasks take as their starting point raw text rather than text that has been correctly segmented into sentences and tokens. It is thus possible for the boundaries of sentences and tokens in the analyses predicted by the systems to differ from those in the gold data. To account for such differences, for all the metrics discussed above, a correct prediction is only measured for predicted tokens that exactly match gold tokens, and performance is measured in terms of precision and recall, the ratio of correct predictions to the number of predicted and gold tokens (respectively). These results are then summarized using the $F_1$ score, the balanced harmonic mean of precision and recall. The quality of the sentence segmentation and tokenization are evaluated using the Sentences and Tokens metrics, which similarly measure the precision, recall, and $F_1$ score for predicting the boundaries of sentences and tokens.

## Results

We next present the results of our experimental evaluation, first comparing the performance of the previous iteration of our system with the newly proposed version with initialization using the various BERT models, and then contrasting the performance of the best-performing variant with previous results on the CRAFT-SA task.

Table 3 summarizes results for variations of our pipeline for all relevant metrics implemented in the evaluation script. To focus on the impact of the model, we here applied gold sentence segmentation and tokenization rather than predicted segmentation. We find that replacing the core parsing components with UDify initialized with the BioBERT model achieves the best results for all but one metric, ranking 2nd on Lemmas by the trivial margin of 0.01% point.

We note that BERT-Large outperforms the Base models mBERT, BlueBERT, and SciBERT across nearly all metrics (again falling trivially behind SciBERT on Lemmas), showing that the benefits of a large model can outweigh those of in-domain training. UDify initialized with BioBERT, which features in-domain training data as well as a Large model shows remarkably strong performance, notably improving on the previous iteration of our parser in the key LAS metric by 2% points over the already very strong result of 90.28%, reflecting an over 20% relative reduction in LAS error. Based on these results, we focused on the variant using UDify initialized with the BioBERT model in our comparison against previous results using predicted segmentation.

Perhaps somewhat surprisingly, we find that UDify initialized with BlueBERT fails to outperform the “general English” Base mBERT model as well as the BERT-Large model and the previous version of our neural parser. This is likely at least in part due to the combination of BlueBERT being a Base model in size (although this holds also for mBERT) and the fact that it is trained on a comparatively smaller corpus (see Table 2). Our results demonstrate that the choice of the pre-trained model is important for achieving state-of-the-art performance and that in-domain pre-training does not guarantee competitive results.

Table 4 contrasts the performance of the newly proposed variant of our parser using UDify and BioBERT initialization (Ours) on the CRAFT-SA test data with raw text input, matching the original shared task evaluation setup. We find again a notable improvement in LAS performance over the previous state of the art, with a 1.5% point difference (approx. 15% reduction in error), confirming the advantage that this model has over the former iteration of the system in terms of core parsing performance. This is reflected also in the MLAS results, which incorporate also information on the performance on predicting part-of-speech tags and morphological features, as well as on the BLEX metric, which requires correct lemmas.

## Discussion and conclusions

In this paper, we have proposed and evaluated a number of approaches incorporating the latest advances in deep transfer learning using contextualized models and neural parsing to dependency parsing of biomedical text. We found that by incorporating the recent UDify neural parser building on the BERT model and initialization using the in-domain Large BioBERT model, the performance of our neural parser on the CRAFT-SA task data could be substantially improved, achieving a LAS of 91.2% and thus reducing LAS error by approximately 15% from the best result achieved in the original shared task. A comparison of various pre-trained BERT models also found that both a Large BERT model as well as appropriate in-domain training data are key to allowing competitive performance at the task and demonstrated the use of a parser and parsing task as a criterion for evaluating and choosing between different BERT models.

Deep transformer-based models such as BERT were introduced comparatively recently, and much of their potential for natural language processing in general and specific tasks such as dependency parsing for biomedical text remains unexplored. A natural extension of the efforts building up to our work would be to explore the use of alternative transformer-based and other deep learning models as well as ways of combining state-of-the-art models and adapting them to better handle texts in the biomedical and other specialized domains. In addition to high-quality pre-trained models, the success of deep transfer learning approaches such as ours also depends critically on the data used for fine-tuning. We have here focused exclusively on the CRAFT corpus syntactic annotation for fine-tuning data, but believe that there remains potential for further improving parsing performance by combining this corpus with other syntactically annotated biomedical and general domain resources, a suggestion we raised also in our previous work [18]. We hope to explore these and related avenues in future work.

We release the newly improved system and all models introduced in this study under open licenses from https://turkunlp.org/Turku-neural-parser-pipeline/models#craft.

## Data Availability

The CRAFT corpus data and the pre-trained models used in this study are available as identified in the relevant sections above, under open licenses. The models newly introduced in this study are available under open licenses from https://turkunlp.org/Turku-neural-parser-pipeline/models#craft.
